# Supplementary material for: Data on DNA-seq analysis of Endophytic Streptomyces sp. SUK 48
Source: Data Brief. 2021 Jan 20;35:106768. doi: 10.1016/j.dib.2021.106768 (PMC7873656; doi:10.1016/j.dib.2021.106768)
Supplement: Supplementary file 1 [file mmc1.pdf]

Data on DNA-seq analysis of Endophytic Streptomyces sp. SUK 48

by Noraziah Mohamad Zin

Submission date: 17-Dec-2020 05:20PM (UTC+0800)
Submission ID: 1477577958
File name: a_on_DNA-seq_analysis_of_Endophytic_Streptomyces_sp._SUK_48.docx (51.1K)
Word count: 1709
Character count: 9728

Data on DNA-seq analysis of Endophytic *Streptomyces* sp. SUK 48

Siti Junaidah Ahmad[1,2], Noraziah Mohamad Zin[1,*]

[1]Center for Diagnostic, Therapeutic and Investigative Studies, Faculty of Health Sciences, Universiti Kebangsaan Malaysia, Jalan Raja Muda Abd Aziz, 50300 Kuala Lumpur, Malaysia.

[2]Faculty of Health Sciences, Universiti Sultan Zainal Abidin, 21300, Kuala Nerus, Terengganu, Malaysia.

*corresponding author: noraziah.zin@ukm.edu.my

Abstract

The data genome sequence of SUK 48 consists of 8,341,706 bp, comprising of one contig with a high G + C content of 72.33%. The genome sequence encodes for 67 tRNAs and 21 rRNAs in one contig. SUK48 was found to have low similarities with other *Streptomyces* sp. (81-93% ANI indices) indicating that the isolated strain has a unique genome property and is presumably a novel species. This genome includes 35 genetic clusters responsible for the synthesis of secondary metabolites, including two polyketide synthase (PKS) clusters; one PKS type II cluster gene, one PKS gene cluster type III, five NRPS genetic clusters, and five PKS/NRPS hybrid clusters. In conclusion, we believe that *Streptomyces* sp. SUK 48 is a novel bacterial species.

Keywords: *Streptomyces* sp., ethnomedicinal plant, whole genome sequence, SUK 48

Specification table

Subject	Biology
Specific subject area	Microbiology, Bacteria genomics, Biotechnology
Type of data	Figure, Table, Draft genome sequence, raw sequence data
How data were acquired	anti-plasmodial activity, genome sequencing by PacBio RS II
Data format	Raw and analyzed
Parameters for data collection	PACBio RS II carried out genome sequencing of the strain. BUSCO annotated and analysed genes; the average Nucleotide Identity (ANI) analysis was also determined. The AntiSMASH software predicted putative biosynthetic gene clusters.
Description of data collection	Endophytic *Streptomyces* sp. SUK 48 was isolated from fruit of *Brasilia* sp. located at the Universiti Kebangsaan Malaysia reserve forest. *Streptomyces* sp. SUK 48 was cultured on Starch Yeast Casein Agar (SYCA) and maintained on International *Streptomyces* Project 2 agar (ISP2) for 14 days on 28°C until whitish spore formed. *Streptomyces* SUK 48 sp. genomic DNA was sequenced by PacBio RS II. Using the NCBI Reference Sequence (RefSeq) protein database and the Swiss-Prot protein database, the functional annotation has been determined. AntiSMASH programme predicted biosynthetic clusters, and genomic data were compared with other Streptomyces spp using ANI (Average Nucleotide Index) analysis.
Data source location	Novel Antibiotic Laboratory, Centre of Diagnostics, Therapeutics & Investigations, Faculty of Health Sciences, Universiti Kebangsaan Malaysia
Data accessibility	The data for this draft genome has been deposited in the GenBank under the accession number CP045740 and the raw reads data under the SRA accession number SRP229701.<br>The data described in this paper is the under bioproject; PRJNA587018 and available at http://www.ncbi.nlm.nih.gov/bioproject/587018.

Value of data:

- *Streptomyces* sp. SUK 48 isolated from *Brasilia* sp. fruit that was sampled along the roadside at Universiti Kebangsaan Malaysia reserve forest
- Towards Streptomyces sp. SUK48 genome, 35 secondary metabolite putative
  - biosynthetic gene clusters were identified
- Data presented in this report could enrich the database of biosynthetic gene clusters in *Streptomyces* bacteria.

1. Data

Here we represent raw data sequence-reads, an assembled data genome of *Streptomyces* sp. SUK 48 isolated from fruit of *Brasilia* sp. Both the raw data and assembled data genome are available at NCBI's Sequence Read Achive as bioproject PRJNA587018 and available at http://www.ncbi.nlm.nih.gov/bioproject/587018.

The predicted coding sequences (≥99 nucleotide) were used for functional annotation. Diamond v0.9.22 was used to BLAST the mRNA sequences against the RefSeq database, while NCBI-Blast v2.2.28+ was used to BLAST the same gene collection against the Swist-Prot database. The cut-offs were set at the overall estimated value of 1 x 10^{-5} for both BLAST searches. For the standalone analysis of the Blast2GO pipeline, the BLAST outputs of both databases were used in gene ontology (GO) and Kyoto Encyclopedia of Genes and Genomes (KEGG) [1]. Secondary metabolism was analyzed via antiSMASH v.5.0 [2].

The analyses of the assembled genome revealed a genome size of about 8,341,706 bp which consisted of 1 contig and with a high G + C content of 72.33% (Table 1). Prior to gene prediction, 67 tRNAs and 21 rRNAs (7 copies of 5S, 7 copies of 16S and 7 copies of 23S)

were identified. A total of 7,354 coding genes were predicted for the masked genome. The gene coding sequences cover approximately 87.7% of the entire draft genome (Table 1). The average nucleotide identity (ANI) analysis of SUK48 with closely related species revealed the following similarity indices: *Streptomyces griseofuscus* 64 (93.85%); *Streptomyces misionensis* DSM 40306 (89.37%); *Streptomyces roseochromogenus* (86.14%); *Streptomyces kebangsaanensis* SUK 12 (84.15%); *Streptomyces coelicolor* A3(2) (83.64%); *Streptomyces exfoliates* NRRL B-2924 (81.48%) and *Streptomyces griseolus* NRRL B-2925 (81.06%). This result suggests that *Streptomyces* sp. SUK 48 strain has unique genome properties.

A total of 7,354 protein coding genes were predicted, approximately 87.7% of the entire draft genome. Of the 7,354 protein coding genes, 7,261 (98.74%) of the genes were annotated with hits using the RefSeq database, whereas BLAST search against Swiss-Prot returned 4,434 or 60.29% of genes with hits. A total of 1,122 putative enzymes were mapped to 139 KEGG pathway maps. About 70.75% (5,203) of the sequences was annotated with 11,887 unique GO identifications. GO (level 2) categories distribution of SUK 48 is briefly described in Figure 1.

In addition, SUK 48 could produce important secondary metabolites . It was estimated that 35 gene clusters will be involved in the secondary metabolism of antiSMASH (Table 2). The present research based on nonribosomal peptides, ectoin and various BGC polyketides (types I, II and III) (biosynthetic gene clusters). While several BGCs were highly homologous with known secondary metabolite synthesis genes such as albaflavenone, ectoine, geosmin and filipine, most of them shared very low similarity with known BGCs. SUK 48 BGCs share the highest gene cluster similarity with the hopene (92 %) of *Streptomyces coelicolor* A3(2). Some NRPS BGCs are preserved and are not closely related to characterised homologs compared to recognised BGCs in the antiSMASH database. For example, kirromycin BGC in regions 12

and 34 only matched by 3-16% to homolog BGCs in SUK 48. This kirromycin BGC had responsible in production of kirromycin which in turn act as anti-plasmodial agent [3].

2. Experimental design, materials and methods

Endophytic *Streptomyces* sp. SUK 48 was isolated from the Universiti Kebangsaan Malasia reserve forest [4]. Fruit of Brasilia sp. was cut into small pieces measured between 3 to 5 cm and cleaned under running tap water to cleanse from macroscopic foreign substance. Then, sterilization steps were done to cleanse form epiphyte microorganism. Sterilization begin with dissolved fruit sample withb 99% ethanol (v/l) within 60 sec. Then, emersed them in 3.5% sodium hypochoride (NaClO) (v/l) within 6 min. Then the sample dissolved back in 99% ethanol (v/l) within 3 to 5 min. Lastly, the sample was rinse three time using sterile distilled water. Sterilization effectiveness was examined through dropping few drops of final sterile water on nutrient agar and incubated the culture at 37 °C for 5 to 7 days. The sample of fruit was culture on AIA (Actinomycetes Isolation Agar) , WA (water agar) and SYCA (Starch Yeast Casein Agar) at 28 ºC and monitored for 7 till 21 days. The bacterium of SUK 48 was isolated after two weeks of culture on SYCA. Then, the culture was maintained on International *Streptomyces* Project 2 agar (ISP2) at 28 ºC [5].

Genomic DNA was extracted by using a Wizard® Genomic DNA Purification Kit as described by the manufacturer (Promega, USA). The sequencing was performed on a PacBio RS II platform (Treecodes, Singapore) generating one SMRT (single-molecule real-time) cell of sequencing data. Briefly, a DNA template consisting of a single molecule bound to a DNA polymerase was immobilized at the bottom of a ZMW (zero mode waveguide). This combined structure was illuminated from below by a laser light. Each of the four DNA bases was connected to one of four different fluorescent colours. When the nucleotide was incorporated into the DNA polymerase, the fluorescent tag was sealed off and diffused out of the ZMW observation field, where its fluorescence was no longer detectable. The detector was used to detect the fluorescent signal of nucleotide incorporation, and the base call was based

on the corresponding fluorescence of the dye [6]. The sequencing data were pre-processed, de novo assembled and polished using the command line pbsmrtpipe of SMRT Link v6.0.0. Then, the Hierarchical Genome Assembly Process 4.0 (HGAP 4.0) was used to assemble the whole genome [7]. The assembly was improved by Quiver iteratively for three times using the resequencing pipeline. Through the Benchmarking Universal Single-Copy Orthologs (BUSCO) assessment, the polished genome was analyzed to obtain the complete genome [6].

The RS II data obtained in the h5 format were converted to the subreads.bam format to be fed into the SMRT Link v6.0.0 [7]. All files in the bax.h5 format were used to create the subreadset.xml file required for the SMRT Link analysis using the –type HdfSubreadSet parameter. The pipeline ID of the pbsmrtpipe.pipelines.sa3_hdfsubread_to_subread was used to convert the h5 reads to the analysis-ready subreads in the subreads.bam format. Next, the subreads were assembled using the Hierarchical Genome Assembly Process 4.0 (HGAP 4.0) pipeline with the pbsmrtpipe.pipelines.polished_falcon_fat. The parameters used for the genome assembly included the following settings: falcon_ns.task_options.HGAP_GenomeLenght_str to 8,000,000, input pa_DBsplit_option=-x500 –s100; ovlp_DBsplit_option = -x500 –s100 for falcon_ns.task_options.HGAP_FalconAdvanced_str along with the aggressive assembly mode turned on. The assembled genome from HGAP 4 was improved by Quiver iteratively for three times using the Resequencing pipeline pbsmrtpipepipeline.sa3_ds_resequencing_fat with default parameters. Benchmarking Universal Single-Copy Ortologs (BUSCO) v2 was used to test the completeness of the polished genome [8] The actinobacteria odb9 profile was selected as the reference profile for this study.

The polished genome was taken as the input for structural annotation. First, tRNA was predicted using tRNAscan-SE v1.3.1 with default parameters [9]. Then, rRNA prediction was carried out using rnammer v1.2 by adding these parameters –S bac and –multi [10].The

polished genome was then masked off for the regions predicted to be tRNAs and rRNAs. The masked polished genome was used for gene prediction using Prodigal v2.6.3 with –c –m turned on. After gene prediction, the full repertoire of peptide sequences (≥ 33 amino acids) was evaluated using BUSCO v2.0. The actinobacteria odb9 profile was selected as the reference profile for this study. The average nucleotide identity (ANI) analysis was calculated according to Goris et al. (2007)[11].

Data on DNA-seq analysis of Endophytic Streptomyces sp. SUK 48

ORIGINALITY REPORT

19%	4%	16%	3%
SIMILARITY INDEX	INTERNET SOURCES	PUBLICATIONS	STUDENT PAPERS

PRIMARY SOURCES

1	Chien-Yeong Wee, Amin-Asyraf Tamizi, Nazrul-Hisham Nazaruddin, Siuk-Mun Ng, Jia-Shiun Khoo, Rosliza Jajuli. "First Draft Genome Assembly of the Malaysian Stingless Bee, Heterotrigona itama (Apidae, Meliponinae)", Data, 2020<br>Publication	7%
2	Xiaoguang Li, Zhikuan Wang, Longzhan Gan, Yongqiang Tian. "Draft genome sequence of Actinocorallia populi A251T, an actinomycetes producing polyketides and nonribosomal polypeptides", 3 Biotech, 2020<br>Publication	3%
3	Quan Dang Nguyen, Phung Minh Truong, Thao Nguyen Thanh Vo, Truc Dao Xuan Chu, Chuong Hoang Nguyen. "Draft genome sequence data of Streptomyces sp. SS1-1, an endophytic strain showing cytotoxicity against the human lung cancer A549 cell line", Data in Brief, 2020<br>Publication	2%

4	Submitted to Deakin University<br>Student Paper	2%
5	archimer.ifremer.fr<br>Internet Source	2%
6	Shasha Wang, Lijing Jiang, Qitao Hu, Xuewen Liu, Suping Yang, Zongze Shao. " Elemental sulfur reduction by a deep-sea hydrothermal vent Campylobacterium sp. ", Environmental Microbiology, 2020<br>Publication	1%
7	bmcmicrobiol.biomedcentral.com<br>Internet Source	1%
8	przeworskilab.com<br>Internet Source	1%
9	nutriweb.org.my<br>Internet Source	1%
10	Wioletta Czaja, Douda Bensasson, Hyo Won Ahn, David J. Garfinkel, Casey M. Bergman. " Evolution of Ty1 copy number control in yeast by horizontal transfer of a gene ", Cold Spring Harbor Laboratory, 2019<br>Publication	<1%

Exclude quotes On

Exclude matches Off

Exclude bibliography On
